# Comprehending phenotypic plasticity in cancer and evolution

**DOI:** 10.1016/j.isci.2024.109308

**Published:** 2024-03-05

**Authors:** Prakash Kulkarni, Ravi Salgia

**Affiliations:** 1 Department of Medical Oncology and Therapeutics Research, City of Hope National Medical Center, Duarte, CA, USA; 2 Department of Systems Biology, City of Hope National Medical Center, Duarte, CA, USA; 3 Division of Biology and Biological Engineering, California Institute of Technology, Pasadena, CA, USA

## Abstract

Organisms as well as cancer cells are adept at adapting to changes in the environment in which they find themselves, by actively adjusting their phenotype. Phenotypic plasticity is a quantitative trait that confers a fitness advantage to the organism by tailoring its phenotype to environmental circumstances. While it is generally held that new traits arise solely from genetic factors, emerging evidence indicates that phenotypic plasticity also plays a critical role both in cancer and evolution. Thus, understanding the mechanisms that underlie phenotypic plasticity can not only provide new insights into organismal evolution and the origin of novelty but can also result in novel strategies and therapeutics to treat cancer.

## Main text

**Figure:**
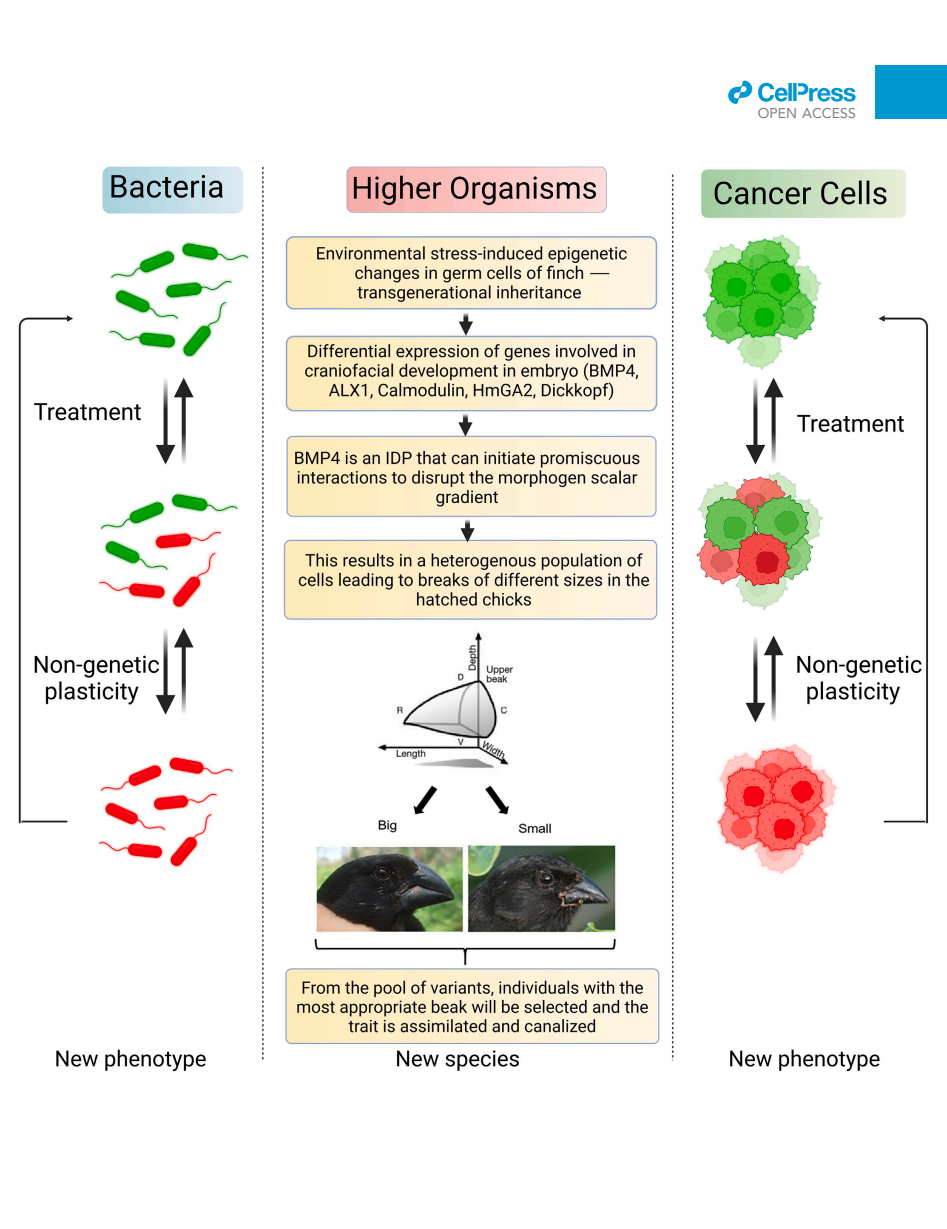
Above image: Phenotypic plasticity in cancer and evolution.

Phenotypic plasticity is the ability of individual genotypes to produce different phenotypes (phenotypic switching) in response to changes in the environment without involving genetic changes.

Research in the field is burgeoning and an often-asked question is whether environmentally initiated phenotypic plasticity precedes and even facilitates adaptation, whether in evolution or in cancer.

It seems obvious that although how populations of organisms and cancer cells respond to modified environmental conditions and adapt themselves is critical to their persistence, the role of phenotypic plasticity in adaptive evolution remains contentious.

While some evolutionary biologists argue that phenotypic plasticity can impede adaptation by shifting the distribution of phenotypes in the population and hence, shielding it from natural selection, others believe phenotypic plasticity favors adaptive evolution.

### Introduction

Phenotypic plasticity is the ability of individual genotypes to produce different phenotypes (phenotypic switching) in response to changes in the environment without involving genetic changes. Plasticity is a quantitative trait that can provide a fitness advantage and mitigate negative effects caused by environmental perturbations. Research in the field is burgeoning and an often-asked question is whether environmentally initiated phenotypic plasticity precedes and even facilitates adaptation, whether in evolution or in cancer. While it is generally held that new traits arise solely from genetic factors, emerging evidence indicates that heritable phenotypes can also arise via non-genetic mechanisms. Further, it is now unequivocal that phenotypic plasticity is ubiquitous in cancer, contributing to disease progression, metastasis, and the emergence of persisters that contribute to heterogeneity in the population. Persistence is a bet-hedging strategy to ensure at least some individuals in a population can survive adverse events. Persisters are believed to give rise to drug resistance.

Indeed, in the revised version of the ‘Hallmarks of Cancer’, phenotypic plasticity and non-genetic mechanisms are identified as the two new hallmarks of cancer.^1^ Although not explicitly mentioned in 1, the term “non-genetic mechanisms” typically implies epigenetic modifications to chromatin. However, new research indicates that the definition should be expanded to also include protein conformational dynamics-based mechanisms.

Furthermore, phenotypic plasticity is also being increasingly recognized as playing an important role in major evolutionary transitions, such as multicellularity. In light of these developments, it seemed prudent to have an in-depth discussion regarding the scope of the phenomenon and its biological implications.

### The genesis of the idea for a thematic meeting and iScience Special Issue

More than a decade ago, the conformational noise hypothesis was enunciated describing how noise emanating from conformational dynamics of intrinsically disordered proteins (IDPs) which is distinct from transcriptional noise, can contribute to phenotypic switching and hence, phenotypic plasticity, by rewiring the cellular protein interaction network.^2^^,^^3^ Since most transcription factors are IDPs, it was posited that conformational noise is an integral component of transcriptional noise implying that IDPs may amplify total noise in the system either stochastically or in response to environmental changes. This concept helped lay the foundation for the genetic/non-genetic duality elaborated by Salgia and Kulkarni^4^ which is now well accepted in the field.^5^^,^^6^^,^^7^ These exciting developments led to the idea of having a meeting focused on phenotypic plasticity in cancer and in evolution.

Thus, the “*Kate and Robert Henderson Colloquium on Phenotypic Plasticity, Cancer, and Evolution*”, was held in November 2023 in Monrovia, California. The two major goals of the meeting were (1) discuss advances in understanding phenotypic plasticity in cancer, and evolution at a system level and (2) translate this knowledge to develop therapeutic approaches to overcome metastasis and drug resistance in cancer. The meeting which included physicists, mathematical biologists, structural and computational biologists, bioinformaticians, eco-devo-evo biologists, cancer biologists, and medical oncologists, was organized in multiple sessions to discuss the various facets of phenotypic plasticity. The sessions were focused on the role of phenotypic plasticity in evolution and in cancer, a quantitative perspective on genetic and non-genetic mechanisms, mathematical modeling to understand deterministic and stochastic events, and protein conformational dynamics or molecular heterogeneity contributing to ‘rewiring’ of the cell’s protein interaction network to actuate phenotypic switching. Pursuant to the meeting, the organizers, Dr. Kulkarni and Dr. Salgia, with help from iScience Editor, Dr. Abul Arif, decided to bring out an *iScience* Special Issue focused on phenotypic plasticity, evolution, and cancer, and also serve as its Guest Editors.

### Phenotypic plasticity and evolution: The puzzling debate

Although it seems obvious that how populations of organisms and cancer cells respond to modified environmental conditions and adapt themselves is critical to their persistence, the role of phenotypic plasticity in adaptive evolution remains contentious. For instance, although it is generally held that phenotypic plasticity can positively impact organism survival under adverse conditions, a general agreement as to whether or not plasticity can drive the evolution of novel traits, and promote taxonomic diversity, is lacking. Likewise, whether phenotypic plasticity plays a role in accelerating or retarding evolutionary change still remains an unsettled issue.^8^

While some evolutionary biologists argue that phenotypic plasticity can impede adaptation by shifting the distribution of phenotypes in the population and hence, shielding it from natural selection, others believe phenotypic plasticity favors adaptive evolution. For example, a recent study^9^ on digital evolution experiments that examined the evolutionary histories of both plastic and non-plastic populations found that, in non-plastic populations, repeated selective sweeps drive the loss of beneficial traits but promote accumulation of maladaptive alleles. However, phenotypic plasticity was observed to stabilize populations against environmental fluctuations thus allowing plastic populations to retain novel adaptive traits than their non-plastic counterparts more easily. Overall, the study found that the stabilizing effect of phenotypic plasticity plays an important role in subsequent adaptive evolution.^9^

Until recently, the concept of phenotypic plasticity remained even more contentious in cancer because of the underlying non-genetic nature of the phenomenon. However, it is now unequivocal that plasticity plays an important role in tumor progression, metastasis, and the evolution of drug resistance, which are fueled by multiple genetic and non-genetic mechanisms. Furthermore, interactions between cancer cells and the components of the tumor microenvironment also influence the fitness of tumor cells. These interactions necessitate changes in the group behavior of tumor cells such as competition and cooperation, highlighting another aspect of plasticity. Phenotypic plasticity and dynamic, non-genetic intra-tumor heterogeneity can modulate both the trajectory of disease progression and adaptive treatment response in cancer.^10^

### System biology and phenotypic plasticity

Gene regulatory networks in an organism, in conjunction with myriad overlapping and interacting homeostatic mechanisms, ensure that many phenotypes are robust to genetic and environmental variation. Often times, modifications of existing gene regulatory circuits drive several important evolutionary adaptations. However, since natural selection acts on phenotypes, a central yet nettlesome question is how selection acts on the genome if the genotype is uncoupled from the phenotype by robustness (canalization) and plasticity mechanisms.

**Figure:**
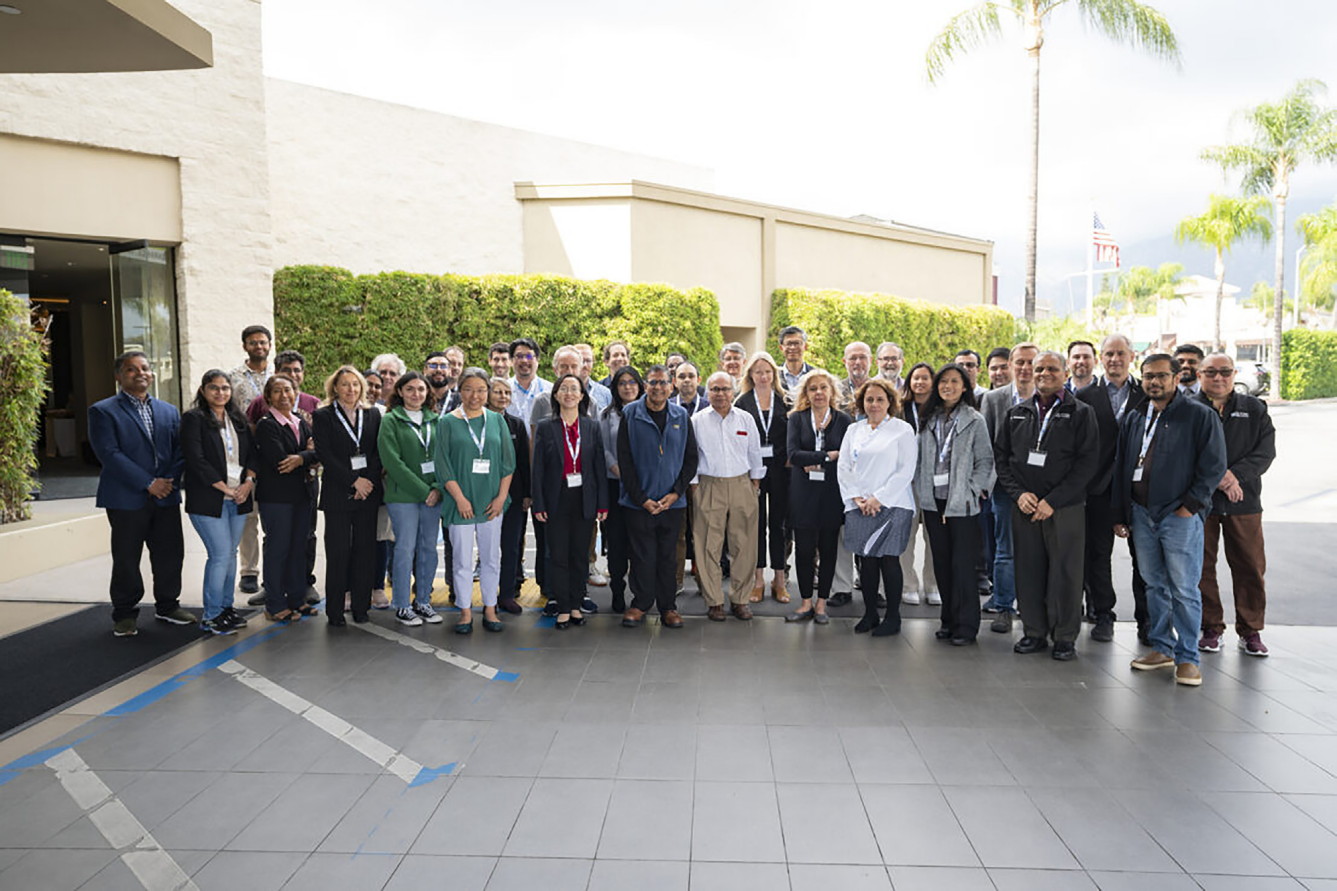
Above image: Attendees of the 1st Kate and Robert Henderson Colloquium on Phenotypic Plasticity, Cancer, and Evolution, held in Monrovia, CA.

System biology approaches have provided new insight into this aspect. Such studies revealed that homeostatic mechanisms operate over a limited range. Beyond that range, the controlled variable changes rapidly allow natural selection to act. Further, mutations and other environmental stressors can disrupt homeostatic mechanisms exposing cryptic genetic variation and allowing natural selection to act.^11^ However, such approaches have also revealed that phenotypic plasticity can result from non-genetic perturbations that include transcriptional and protein conformational noise, especially from IDPs that occupy key positions in cellular protein interaction networks, as well as from epigenetic modifications.^3^

However, since plasticity is not infinite, there may be inherent costs involved that are significant. Theoretical studies to analyze a model system of plastic development and derive its limits to promote population growth identified an explicit expression for growth rate which can be decomposed into several easily interpretable terms that represent the benefits and limitations of plasticity as an adaptation strategy. Furthermore, the expression takes a form that is remarkably similar to that obtained for bet-hedging strategy whereby a population stochastically assumes phenotypic heterogeneity, underscoring how evolutionary strategies may be unified under a common general framework employing system biology.^12^

In cancer too, where phenotypic plasticity can lead to complex cell state dynamics during disease progression and emergence of drug resistance, system biology approaches have helped identify high-plasticity states and elucidate transition paths between states. Epithelial to mesenchymal transition (EMT) is a case in point.^13^ Further, the identification of hybrid epithelial/mesenchymal states/phenotypes in EMT that appears to be a prerequisite for tumor metastasis, is a good example.^14^ Such approaches have yielded novel insights into cancer metastasis which requires tumor cells to be proliferative and invasive at the same time. Again, system biology-based approaches have revealed how tumor cells compensate for this trade-off by changing their phenotype during metastasis through phenotypic plasticity. An eco-evo-inspired mathematical models suggest that phenotypically plastic tumor cell populations attain a stable phenotype equilibrium that maintains tumor cell heterogeneity, paving the way for new therapeutic treatment strategies such as ‘adaptive’ or intermittent therapy.^15^

### What the future holds

The colloquium highlighted the power of a system biology approach integrating empirical observation and abstraction. The take-home message from this exciting meeting that reverberated with highly illuminating and eloquent presentations and passionate and, at times, enthralled discussions, was that phenotypic plasticity is fundamental to both evolution and cancer. However, it was also clear that many important questions remain unexplored. For example, can we gain deeper insight into cell fate specification employing dynamical systems theory? Can this provide a conceptual framework to better understand how attractors decide cell fate so that this information may be used to ‘cajole’ cancer cells to shift to ‘normal’ attractors? How does molecular heterogeneity i.e., protein conformational dynamics, translate to phenotypic heterogeneity via cellular protein interaction network rewiring? Is the information generated by integrating external cues transmitted to the genome so that it can be inherited transgenerationally? Does it involve epigenetic modifications to the genome? How does the cell “decide” when and where to make such modifications, and how are they retained during development? With regard to the genetic/non-genetic duality, are these mutually exclusive or do they act in unison with reversible non-genetic mechanisms invoked first followed by genetic mechanisms (mutations/alterations) that are irreversible? Do mutations occur randomly, and do appropriate ones get selected, or do they predominantly occur in certain preferred locations (“hot spots”) in genes encoding certain proteins that occupy critical (e.g., hub) positions in the protein interaction network to relieve network frustration?

There is now emerging evidence that transition/hybrid phenotypic states exist. It will be interesting to know if these are functional and advantageous or, are they merely artifacts discerned by the observer. If transition/hybrid states genuinely exist, do they reflect a bet-hedging strategy? Does noise play a role in generating phenotypic heterogeneity? Does the group behavior of individuals in a population contribute to heterogeneity? Are there parallels between cancer and evolution that can explain and delineate phenotypic plasticity?

The answers to these questions will undoubtedly provide deeper insights into organismal evolution. Furthermore, discerning context-specific cancer cell states and the molecular mechanisms regulating them could help identify new treatments for cancer underscoring the ethos of the Henderson Colloquium. The success of the meeting further underscored the need for more future meetings focused on phenotypic plasticity, cancer, and evolution, with the fond hope that the meetings will result in novel strategies and therapeutics to treat cancer.
